# Supplementary material for: The exact phase diagram for a class of open multispecies asymmetric exclusion processes
Source: Sci Rep. 2017 Oct 19;7:13555. doi: 10.1038/s41598-017-12768-8 (PMC5648981; doi:10.1038/s41598-017-12768-8)
Supplement: Supplementary file 1 — Supplementary Information: The exact phase diagram for a class of open multispecies asymmetric exclusion processes [file 41598_2017_12768_MOESM1_ESM.pdf]

SUPPLEMENTARY INFORMATION: THE EXACT PHASE DIAGRAM FOR A CLASS OF OPEN MULTISPECIES ASYMMETRIC EXCLUSION PROCESSES

ARVIND AYYER AND DIPANKAR ROY

I. The semipermeable TASEP

We review the semipermeable TASEP [1] using the original terminology, but with the notation of the mTASEP. It was originally conceived as a model of first-class particles (1's), second-class particles (0's) and vacancies ($\overline{1}$'s) where both particles hop only to the right, and where first-class particles overtake second-class ones. In addition, 0's are constrained to remain in the system. This model then corresponds to the mTASEP with $r = 1$. We consider the large-size limit of the semipermeable TASEP so that the ratio of the number of 0's to the size of the system approaches a fixed constant, θ_0. The phase diagram is thus determined by three parameters: α, β and θ_0.

The phase diagram of the semipermeable TASEP was understood using two important concepts. The first was a *current-density relation*, which was derived assuming the typical configuration in the stationary distribution has uniform regions of a given density. This showed that either $\rho_1 = \rho_{\overline{1}}$ or $\rho_1 = 1 - \rho_{\overline{1}}$ in the bulk. The second was a *colouring argument* which said the following. If one could not distinguish between 0's and $\overline{1}$'s, then the dynamics were the same as the original TASEP in the bulk between 1's and these two. A similar argument was made if one could not distinguish between 1's and 0's. Although these identifications fail at the boundaries, this argument gives the correct physical picture. These two ideas led to the notion of a *fat shock*, a macroscopic region in which all the second class particles are present. The width of the fat shock was then determined by the number of confined 0's.

An illustration of the fat shock is given in Fig. S1. The regions separating $\overline{1} - 0$ and $0 - 1$ execute synchronised random walks so that the width of the fat shock is fixed. In various regions of the phase diagram, the fat shock has either negative, positive, or zero drift.

Since 0's are confined to the lattice, their average current vanishes. Because of particle conservation, the current of 1's to the right is the same as that of $\overline{1}$'s to the left. We will thus describe only the former, denoted J_1. In region $\mathbb{0}$ = C, the fat shock permeates the system and thus, all densities are constant. The density of 1's and $\overline{1}$'s is $\rho_1 = \rho_{\overline{1}} = (1-\theta_0)/2$ and their current is $J_1 = (1-\theta_0^2)/4$. In region $\overline{\mathbb{1}}$ = A, the fat shock is pinned on the right, the density of 1's is $\rho_1 = \alpha$ throughout and the current is $J_1 = \alpha(1-\alpha)$. In region $\mathbb{1}$ = B, the fat shock is pinned on the left, the density of $\overline{1}$'s is $\rho_{\overline{1}} = \beta$ throughout and the current is $J_1 = \beta(1-\beta)$. On the $\overline{\mathbb{1}} - \mathbb{1}$ boundary, the two ends of the shock perform synchronised oscillations so

Date: August 8, 2017.

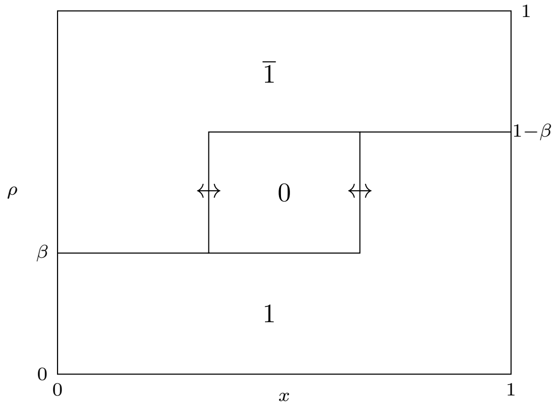

FIG. S1. Instantaneous picture of the fat shock in the $\mathbb{1}-\overline{\mathbb{1}}$ boundary in the semipermeable TASEP. Each connected region is labelled with the species of a particle and the height of a region at a given location represents the density of that species at that point.

as to keep the width fixed. In all regions, the width is governed by the fraction θ_0, which is fixed.

Although the existence of the fat shock was proved rigorously only for the semipermeable TASEP [1], the calculations of the density and current for the semipermeable ASEP in Ref. [5] strongly suggest that they exist in the latter. The simulations in Supplementary Fig. S2 attest to this hypothesis. The formulas for the densities and currents in the semipermeable ASEP are summarised in Supplementary Table S1.

II. Proofs of the results

In this section, we will prove the formulas for the currents and densities in Tables 2 and 1 respectively, from which the phase diagram in Main Fig. 2 will follow. We will first prove the ergodicity of the mASEP with conserved charges $\underline{n} = (n_0, \dots, n_r)$.

Proposition 1. *The mASEP is ergodic.*

Proof. It will suffice to prove that the mTASEP with conserved charges $\underline{n} = (n_0, \dots, n_r)$ is ergodic. This follows from abstract considerations in the theory of affine Coxeter groups [2], but we will prove the ergodicity from first principles.

Define the configurations

$$\begin{aligned} \delta^{\uparrow} &= (\underbrace{0, \dots, 0}_{n_0}, \dots, \underbrace{r, \dots, r}_{n_r}), \text{ and} \\ \delta^{\downarrow} &= (\underbrace{0, \dots, 0}_{n_0}, \dots, \underbrace{\overline{r}, \dots, \overline{r}}_{n_r}), \end{aligned} \tag{S1}$$

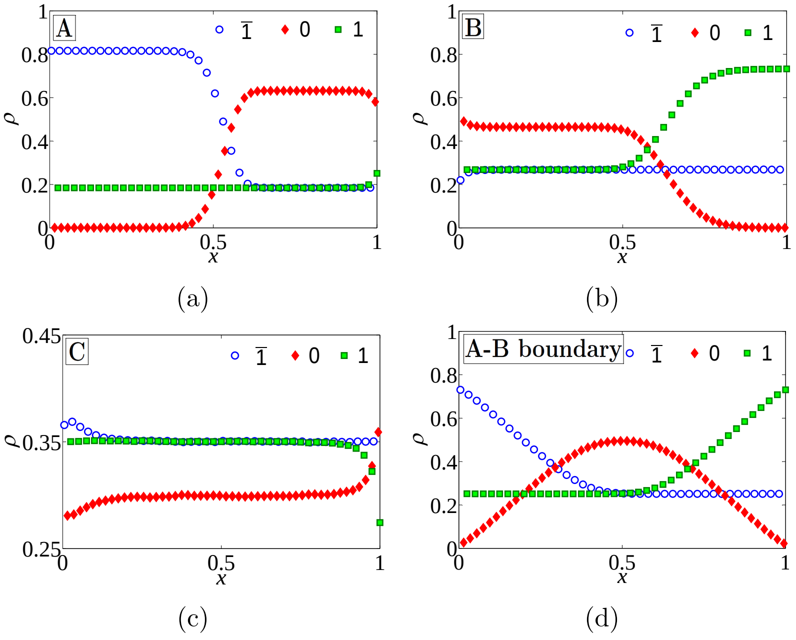

FIG. S2. Plots of the densities of particles 1 (green squares), 0 (red diamonds), and $\overline{1}$ (blue circles), versus the scaled position $x = m/n$ for the $r = 1$ semipermeable ASEP with $n = 200, \theta_0 = 0.3$ and $\theta_1 = 0.7$ in the regions (a) A ($\alpha = 0.19, \beta = 0.78, \gamma = 0.41, \delta = 0.63, q = 0.47$), (b) B ($\alpha = 0.89, \beta = 0.34, \gamma = 0.43, \delta = 0.63, q = 0.59$), (c) C ($\alpha = 0.84, \beta = 0.89, \gamma = 0.61, \delta = 0.43, q = 0.59$), and the (d) A-B boundary ($\alpha = \beta = 0.34, \gamma = \delta = 0.63, q = 0.52$).

both of which belong to the state space. Let $m_j(\tau)$ be the number of j's in τ for $j \in \{\overline{r}, \dots, 0, \dots, r\}$ and $\underline{m} = (m_{\overline{r}}, \dots, m_0, \dots, m_r)$. Define

$$\text{(S2)} \qquad \begin{aligned} \delta^{\uparrow}_{\underline{m}} &= (\underbrace{\overline{r}, \dots, \overline{r}}_{m_{\overline{r}}}, \dots, \underbrace{0, \dots, 0}_{m_0}, \dots, \underbrace{r, \dots, r}_{m_r}), \\ \delta^{\downarrow}_{\underline{m}} &= (\underbrace{r, \dots, r}_{m_r}, \dots, \underbrace{0, \dots, 0}_{m_0}, \dots, \underbrace{\overline{r}, \dots, \overline{r}}_{m_{\overline{r}}}). \end{aligned}$$

We will first show that the following sequence can be obtained

$$\text{(S3)} \qquad \delta^{\downarrow} \longrightarrow \delta^{\downarrow}_{\underline{m}} \longrightarrow \tau \longrightarrow \delta^{\uparrow}_{\underline{m}} \longrightarrow \delta^{\uparrow}.$$

First of all, it is clear that τ can be obtained starting from $\delta^{\downarrow}_{\underline{m}}$ and $\delta^{\uparrow}_{\underline{m}}$ can be obtained from τ by making purely bulk transitions. To make transitions from $\delta^{\downarrow}$ to $\delta^{\downarrow}_{\underline{m}}$, first move m_1 of the $\overline{1}$'s to the left by bulk transitions and convert them to 1's. Repeat this procedure for m_2 of the 2's, and so on, all the way till m_r of the r's. We then end up with $\delta^{\downarrow}_{\underline{m}}$. Finally, starting with $\delta^{\uparrow}_{\underline{m}}$, convert all $m_{\overline{r}}$ $\overline{r}$'s to r's on the left boundary and move them to the right. Continue this way for $(\overline{r-1})$'s and so on, all the way till $\overline{1}$'s, ending up with $\delta^{\uparrow}$.

Region	Densities			Current of 1's
	ρ_1	ρ_0	$\rho_{\bar{1}}$	
$\mathbb{0} =$ C $(a, b < \zeta_0)$	$(1-\theta_0)/2$	θ_0	$(1-\theta_0)/2$	$(1-\theta_0^2)/4$
$\bar{\mathbb{1}} =$ A $(b, \zeta_0 < a)$	$f(a)$	$\begin{cases} 0 & x < x_0 \\ 1-2f(a) & x > x_0 \end{cases}$	$\begin{cases} 1-f(a) & x < x_0 \\ f(a) & x > x_0 \end{cases}$	$(1-q)f(a)(1-f(a))$
$\mathbb{1} =$ B $(a, \zeta_0 < b)$	$\begin{cases} f(b) & x < x_1 \\ 1-f(b) & x > x_1 \end{cases}$	$\begin{cases} 1-2f(b) & x < x_1 \\ 0 & x > x_1 \end{cases}$	$f(b)$	$(1-q)f(b)(1-f(b))$
A-B boundary $(\zeta_0 < a = b)$	$\begin{cases} f(a) & x < x_1 \\ \text{linear} & x > x_1 \end{cases}$	$\begin{cases} 1-2f(a) & x < x_1 \\ & \text{or } x > x_0 \\ \text{linear} & \text{otherwise} \end{cases}$	$\begin{cases} \text{linear} & x < x_0 \\ f(a) & x > x_0 \end{cases}$	$(1-q)f(a)(1-f(a))$

TABLE S1. Densities and currents of the semipermeable ASEP. We have used the notation θ_0 for the fixed fraction of 0's, and set $\zeta_0 = (1+\theta_0)/(1-\theta_0)$, $x_0 = 1 - \theta_0(a+1)/(a-1)$ and $x_1 = \theta_0(b+1)/(b-1)$.

We will now construct a sequence of moves taking $\delta^{\uparrow}$ to $\delta^{\downarrow}$, which is the difficult part of the proof. We will do this in r stages, where the end of the j'th stage will be the configuration given by

$$\sigma_j = (\underbrace{\overline{r}, \dots, \overline{r}}_{n_r}, \dots, \underbrace{\overline{j+1}, \dots, \overline{j+1}}_{n_{j+1}}, \underbrace{0, \dots, 0}_{n_0}, \underbrace{\overline{1}, \dots, \overline{1}}_{n_1}, \dots, \underbrace{\overline{j}, \dots, \overline{j}}_{n_j}). \tag{S4}$$

Starting from $\delta^{\uparrow}$, convert all r's to $\overline{r}$'s and move them to the left. Repeat this procedure for $\overline{r-1}$'s continuing up to $\overline{2}$'s. Now convert all 1's to $\overline{1}$'s to end up with σ_1. We now show how to reach σ_{j+1} from σ_j. First, convert all $\overline{r}$'s to r's, move them to the right, and convert them back to $\overline{r}$'s. Repeat this procedure for $\overline{r-1}$'s continuing up to $\overline{j+1}$'s. Now move all $\overline{r}, \dots, \overline{j+2}$'s to the left using bulk transitions to end up with σ_{j+1}. Once we have repeated this argument r times, we end up with $\sigma_r = \delta^{\downarrow}$.

We have thus shown how to reach $\delta^{\uparrow}$ from τ and vice versa for every configuration τ, thus completing the proof. □

As an illustration of Proposition 1, we give the following example.

Example 2. *The transition graph of the mTASEP with* $r = 2$ *and* $\underline{n} = (1, 1, 1)$ *is given in Supplementary Fig. S3.*

Since the mASEP is ergodic, it possesses a unique nonequilibrium steady state (NESS); see [4, Theorem 20.1], for example.

Remark 1. *The mASEP possesses charge-conjugation symmetry in the following sense: interchanging positively and negatively charged particles as well as the rates* 1 *and* q*,* α *and* β*, and* γ *and* δ*, and changing the direction of motion leaves the mASEP invariant.*

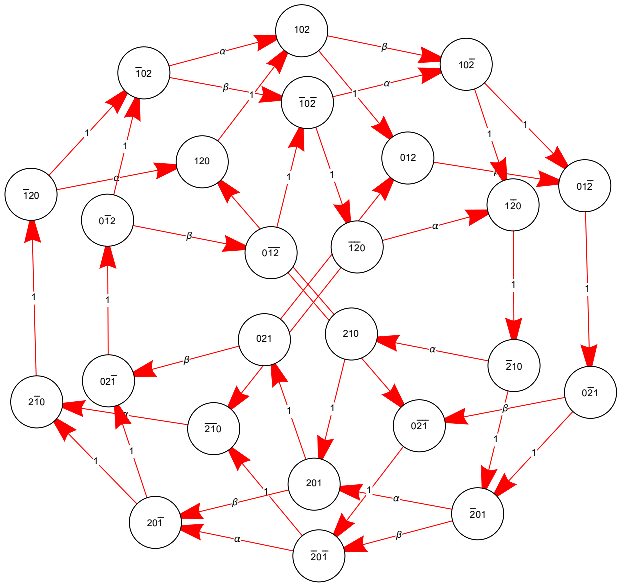

FIG. S3. The transition graph of the mTASEP with $\underline{n} = (1, 1, 1)$. The rates associated to each transition are marked on the arrows.

We will now prove properties of the NESS of the mASEP. The key idea in the proof will be played by a general notion of lumping of Markov processes, which we define here for completeness.

Definition 1 ([4, Section 2.3])**.** *Let (X_t) be a Markov process on a state space Ω with Markov matrix M. Let $\mathcal{S}$ be a set partition of Ω (i.e. a collection of pairwise disjoint subsets of Ω whose union is Ω). If, for all $S, T \in \mathcal{S}$, and $x, x' \in S$,*

$$\sum_{y \in T} M(y, x) = \sum_{y \in T} M(y, x'), \tag{S5}$$

then the stochastic process (Y_t) on $\mathcal{S}$ which is obtained from (X_t) by keeping track of the subset of Ω is itself a Markov process, with Markov matrix M' given by $M'(T, S) = \sum_{y \in T} M(y, x)$. The process (Y_t) is then said to be a lumping *of (X_t).*

Recall that the mASEP is defined by the conserved charges $\underline{n} = (n_0, \dots, n_r)$ with state space $\Omega_{\underline{n}}$.

Definition 2. *For k such that $1 \le k \le r$, set $\underline{n}' = (n_0 + \cdots + n_{k-1}, n_k + \cdots + n_r)$. Define the map $\lambda_k : \Omega_{\underline{n}} \to \Omega_{\underline{n}'}$ as follows:*

$$\lambda_k(\tau_i) = \begin{cases} 0 & \textit{if } \tau_i \textit{ has charge } j \textit{ or } \overline{j} \textit{ and } 0 \le j < k, \\ 1 & \textit{if } \tau_i \textit{ has charge } j \textit{ and } j \ge k, \\ \overline{1} & \textit{if } \tau_i \textit{ has charge } \overline{j} \textit{ and } j \ge k. \end{cases} \tag{S6}$$

Then the k-colouring *of the mASEP with conserved charges* $\underline{n}$ *is the set partition of* $\Omega_{\underline{n}}$ *obtained by* λ_k^{-1}.

Proposition 3. *Let* $\underline{n} = (n_0, \dots, n_r)$ *and* $1 \le k \le r$. *The* k*-colouring leads to a lumping of the mASEP with conserved charges* $\underline{n}$ *onto the semipermeable ASEP with* $n_0 + \cdots + n_{k-1}$ *particles of species* 0.

Proof. One can check that the transitions in the bulk, as well as those in the left and right boundary, commute with the map λ_k. □

As a consequence of Proposition 3, we will call the lumping of the mASEP via the k-colouring as the *k-coloured semipermeable ASEP*. We now obtain the densities of species r and $\overline{r}$ in the mASEP.

Proposition 4. *The densities at site* i *of species* r *and* $\overline{r}$ *(resp. current* J_r *of species* r*) in the mASEP with conserved charges* $(n_0, \dots, n_r)$ *are the same as those of the densities at site* i *of species* 1 *and* $\overline{1}$ *respectively (resp. current* J_1 *of species* 1*) in the semipermeable ASEP with size* $n_0 + \cdots + n_r$ *with* $n_0 + \cdots + n_{r-1}$ *particles of species* 0.

Proof. This follows immediately from Proposition 3 using the r-colouring. □

To arrive at the main result, we take the limit of the mASEP with r species of charges with $n_0, \dots, n_r \to \infty$ such that $n_j/n \to \theta_j$ for all $0 \le j \le r$.

Theorem 5. *The densities and currents of particles of all species in all phases in the mASEP with* r *species of charges are given in Main Table 1 and Main Table 2 respectively.*

Proof. We will simultaneously use all possible k-colourings in the proof. First, we fix some notation. For the k-coloured ASEP, we denote the species by $1^{(k)}, 0^{(k)}$ and $\overline{1}^{(k)}$. The (fixed) number of particles of species $0^{(k)}$ is $n_{0^{(k)}} = n_0 + \cdots + n_{k-1}$ and hence $\theta_{0^{(k)}} = \theta_0 + \cdots + \theta_{k-1}$ and similarly for $\theta_{1^{(k)}}$ and $\theta_{\overline{1}^{(k)}}$. The single-site densities and currents of the k-coloured species are related to those of the original mASEP as follows:

$$
\begin{aligned}
\rho_{1^{(k)}} &= \rho_k + \cdots + \rho_r, \\
\rho_{\overline{1}^{(k)}} &= \rho_{\overline{k}} + \cdots + \rho_{\overline{r}}, \\
\rho_{0^{(k)}} &= \rho_{\overline{k-1}} + \cdots + \rho_{k-1}, \\
J_{1^{(k)}} &= J_k + \cdots + J_r.
\end{aligned} \tag{S7}
$$

We give the proof of the densities and currents in phase $\mathbb{j}$. The argument for the other phases and phase-boundaries is similar. Recall that the phase $\mathbb{j}$ is defined by the constraints

$$
\frac{1 + \theta_0 + \cdots + \theta_{j-1}}{1 - (\theta_0 + \cdots + \theta_{j-1})} < b < \frac{1 + \theta_0 + \cdots + \theta_j}{1 - (\theta_0 + \cdots + \theta_j)} \text{ and } b > a, \tag{S8}
$$

according to the phase diagram in Main Fig. 2. Now, consider the k-colouring of the mASEP for $k \le j$. In that case, we see that $b > (1 + \theta_{0^{(k)}})/(1 - \theta_{0^{(k)}})$ and hence, the k-coloured semipermeable ASEP is in phase $\mathbb{1} = \text{B}$. Thus $\rho_{\overline{1}^{(k)}}$ equals $1/(b+1)$ in the bulk (from Supplementary Table 1) for each $1 \le k \le j$. Therefore,

$$
\rho_{\overline{1}} + \cdots + \rho_{\overline{r}} = \rho_{\overline{2}} + \cdots + \rho_{\overline{r}} = \cdots = \rho_{\overline{j}} + \cdots + \rho_{\overline{r}} = 1/(b+1), \tag{S9}
$$

from which it follows that $\rho_{\overline{1}} = \rho_{\overline{2}} = \cdots = \rho_{\overline{j-1}} = 0$. Now, consider the k-colouring of the mASEP for $j < k \leq r$. In that case,

$$\text{(S10)} \qquad \frac{1+\theta_{0^{(k)}}}{1-\theta_{0^{(k)}}} > \frac{1+\theta_0+\cdots+\theta_j}{1-(\theta_0+\cdots+\theta_j)} > b,$$

and the k-coloured semipermeable ASEP is in $\mathbb{0}$ = C. From Supplementary Table 1, we see that

$$\text{(S11)} \qquad \rho_{\overline{1}^{(k)}} = \frac{1-\theta_{0^{(k)}}}{2} = \frac{\theta_k+\cdots+\theta_r}{2},$$

and hence $\rho_k = \theta_k/2$ for each $j+1 \leq k \leq r$. Now, using (S9), we can calculate the density of $\overline{j}$. We have thus obtained the densities of all negatively charged species of particles. See the top row of Main Fig. 3(a) for comparison. Similar computations yield the densities of positively charged species and 0, as well as the currents. □

In [3, Corollary 2], it is shown that the nonequilibrium partition function $Z_{\underline{n}}$ is given by the following explicit product formula

$$\text{(S12)} \qquad Z_{\underline{n}} = \prod_{k=1}^{r} Z_{(n_0+\cdots+n_{k-1}, n_k+\cdots+n_r)},$$

where $Z_{(n_0,n_1)}$ is the partition function for the semipermeable ASEP [5, Equation (5.6)], where the notation $L = n_0 + n_1$ and $N = n_0$ is used for the system size and the number of 0's respectively. Theorem 5 provides a justification (although not a proof) of why the factorisation of the partition function in (S12) is to be expected. The k'th term on the right is precisely the partition function of the k-coloured semipermeable ASEP explained in Proposition 3.

References

[1] A. Ayyer, J. L. Lebowitz, and E. R. Speer. On the two species asymmetric exclusion process with semi-permeable boundaries. *J. Stat. Phys.*, 135(5-6):1009–1037, 2009.

[2] Anders Björner and Francesco Brenti. *Combinatorics of Coxeter groups*, volume 231 of *Graduate Texts in Mathematics*. Springer, New York, 2005.

[3] Luigi Cantini, Alexandr Garbali, Jan de Gier, and Michael Wheeler. Koornwinder polynomials and the stationary multi-species asymmetric exclusion process with open boundaries. *J. Phys. A*, 49(44):444002, 2016.

[4] David A. Levin, Yuval Peres, and Elizabeth L. Wilmer. *Markov chains and mixing times*. American Mathematical Society, Providence, RI, 2009. With a chapter by James G. Propp and David B. Wilson.

[5] Masaru Uchiyama. Two-species asymmetric simple exclusion process with open boundaries. *Chaos Solitons Fractals*, 35(2):398–407, 2008.

Arvind Ayyer, Department of Mathematics,, Indian Institute of Science,, Bangalore - 560012, India

E-mail address: arvind@iisc.ac.in

Dipankar Roy, Department of Mathematics,, Indian Institute of Science,, Bangalore - 560012, India

E-mail address: dipankarroy@iisc.ac.in
